# Attenuated FOLFIRINOX in the salvage treatment of gemcitabine-refractory advanced pancreatic cancer: a phase II study

**DOI:** 10.1186/s40880-018-0304-1

**Published:** 2018-06-04

**Authors:** Jung Hoon Kim, Sang-Cheol Lee, Sung Yong Oh, Seo-Young Song, Namsu Lee, Eun Mi Nam, Soonil Lee, In Gyu Hwang, Hyo Rak Lee, Kyu Taek Lee, Sang-Byung Bae, Han Jo Kim, Joung Soon Jang, Do Hyoung Lim, Hyun Woo Lee, Seok Yun Kang, Jung Hun Kang

**Affiliations:** 1 Department of Internal Medicine, Institute of Health Sciences, Gyeongsang National University, 15 Jinju-daero 816beon-gil, Jinju, 52727 Republic of Korea; 2 Divsion of Hematology and Oncology, Department of Internal Medicine, Soonchunhyang University Hospital Cheonan, Cheonan, 31151 Republic of Korea; 3 Department of Internal Medicine, Dong-A University Hospital, Busan, 49201 Republic of Korea; 4 Department of Internal Medicine, Kangwon National University School of Medicine, Chuncheon, 24289 Republic of Korea; 5 Divsion of Hematology and Oncology, Department of Internal Medicine, Soonchunhyang University Hospital Seoul, Seoul, 04401 Republic of Korea; 6 Department of Internal Medicine, Ewha Womans University, College of Medicine, Seoul, 07985 Republic of Korea; 7 Department of Internal Medicine, Dankook University Hospital, Cheonan, 31116 Republic of Korea; 8 Department of Internal Medicine, Chung-Ang University, College of Medicine, Seoul, 06973 Republic of Korea; 9 Division of Hematology and Medical Oncology, Department of Internal Medicine, Korea Cancer Hospital, Seoul, 01812 Republic of Korea; 10 Department of Hematology-Oncology, Ajou University Hospital, Suwon, 16499 Republic of Korea

**Keywords:** Attenuated FOLFIRINOX, Second-line, Pancreatic cancer, Gemcitabine

## Abstract

**Background:**

Combination therapy with oxaliplatin, irinotecan, fluorouracil, and leucovorin (FOLFIRINOX) chemotherapy drastically improves survival of advanced pancreatic cancer patients. However, the efficacy of FOLFIRINOX as a second-line treatment after gemcitabine failure has not been tested prospectively. We investigated the feasibility and safety of attenuated FOLFIRINOX in patients with gemcitabine-refractory advanced pancreatic cancer.

**Methods:**

A multicenter phase II prospective open-label, single-arm study was conducted at 14 hospitals. Patients with histologically proven invasive ductal pancreatic adenocarcinoma, a measurable or evaluable lesion, Eastern Cooperative Oncology Group performance status 0 or 1, adequate organ function, and aged 19 years or older were eligible. Attenuated FOLFIRINOX consisted of oxaliplatin 65 mg/m^2^, irinotecan 135 mg/m^2^, and leucovorin 400 mg/m^2^ injected intravenously on day 1 and 5-fluorouracil 2000 mg/m^2^ continuously infused intravenously over 46 h on days 1–2, repeated every 2 weeks. The primary endpoint was progression-free survival from the initiation of FOLFIRINOX. Secondary endpoints were the objective response rate, disease control rate, overall survival, safety, and tolerability. We estimated overall survival and progression-free survival using the Kaplan–Meier methods.

**Results:**

We enrolled 39 patients from 14 institutions. The objective response rate was 10.3%, while the disease control rate was 64.1%. The 6-month and 1-year overall survival rates were 59.0% and 15.4%, respectively. Median progression-free survival and overall survival were 3.8 months (95% confidence interval [CI] 1.5–6.0 months) and 8.5 months (95% CI 5.6–11.4 months), respectively. Grade 3 or 4 adverse events were neutropenia (41.0%), nausea (10.3%), anorexia (10.3%), anemia (7.7%), mucositis (7.7%), pneumonia/pleural effusion (5.1%), and fatigue (5.1%). One treatment-related death attributable to septic shock occurred.

**Conclusion:**

Attenuated FOLFIRINOX may be promising as a second-line therapy for gemcitabine-refractory pancreatic cancer.

## Background

Pancreatic cancer remains an obstinate disease despite recent advances in diagnostic and therapeutic science and techniques. It is the fifth-leading cause of cancer-related deaths in Korea [1, 2], and its 5-year relative survival rate is approximately 7.6% across all stages [3]. Only 10%–20% of patients are diagnosed with resectable disease at presentation [4–6]. A large proportion of patients have advanced disease at initial presentation. Studies aimed at developing an effective systemic treatment for pancreatic cancer have elicited slow but steady advances in survival benefits. The greatest improvement in overall survival (OS) was demonstrated by the randomized phase III PRODIGE 4/ACCORD 11 trial, which revealed that combination therapy with oxaliplatin, irinotecan, 5-fluorouracil, and leucovorin (FOLFIRINOX) was superior to gemcitabine alone [7, 8]. The median progression-free survival (PFS) and OS in the FOLFIRINOX group were 6.4 and 11.1 months, respectively (compared with 3.3 and 6.8 months in the gemcitabine group). The reported objective response rate was 31.6% versus 9.4% (FOLFIRINOX versus gemcitabine) [7].

The trial with FOLFIRINOX was an important milestone in the treatment of pancreatic cancer, although it raised a logical concern regarding toxicity and safety [9]. Grade 3 or 4 neutropenia occurred in 45.7% of patients (75 in 164 patients), and one case of treatment-related death due to febrile neutropenia was reported in the FOLFIRINOX arm, despite the investigators’ more rigorous selection of participating patients than that reported in other studies [7].

A salvage systemic therapy for patients with advanced pancreatic cancer refractory to the frontline regimen had not been established when we started the present study; however, several investigators have, since then, conducted pilot or phase II trials with combinations such as FOLFIRI [10], FOLFOX [11], and IROX [12]. Lee et al. [13] investigated FOLFIRINOX as a second-line chemotherapy in patients with advanced pancreatic cancer who progressed on gemcitabine-based therapy, but the study was a retrospective analysis including only 18 patients. While our study was in progress, the results of a phase III study (CONKO-003 trial) comparing oxaliplatin, folinic acid, and fluorouracil (OFF) with folinic acid and fluorouracil (FF) for gemcitabine-refractory pancreatic cancer were published; this work is the only phase III comparative study to date that has verified survival benefits of second-line chemotherapy for advanced pancreatic cancer [14].

Current guidelines recommend the application of FOLFIRINOX to a highly selective group of patients, such as patients with Eastern Cooperative Oncology Group (ECOG) performance status (PS) of 0 or 1, good pain management, patent biliary stent, and adequate nutritional intake [15]. Gemcitabine-based chemotherapeutic regimens, which are used widely in clinical practice, are still recommended as category 1 for most patients.

Fluoropyrimidine-based chemotherapy is recommended as a second-line treatment for patients who have progressed after gemcitabine-based first-line therapy. However, to our knowledge, no prospective investigation has reported on whether FOLFIRINOX therapy can influence the outcomes of patients who maintain good performance status after gemcitabine failure.

Thus, we conducted a single-arm phase II trial to evaluate efficacy and safety of second-line dose-attenuated FOLFIRINOX for treating gemcitabine-refractory patients with locally advanced unresectable or metastatic pancreatic cancer in which curative therapy was not feasible.

## Patients and methods

### Study design

This trial was a Korean, multicenter phase II prospective open-label, single-arm study.

The primary endpoint of our study was PFS. The definition of PFS was the time from initiation of FOLFIRINOX until confirmation of progressive disease or death. Secondary endpoints were the objective response rate (ORR), disease-control rate (DCR), OS, and safety and tolerability of patients. ORR was defined as the proportion of patients who showed complete response (CR) or partial response (PR), and DCR was defined as the proportion of patients who showed stable disease (SD).

### Patient selection

Adult patients with histologically confirmed pancreatic adenocarcinoma on which curative treatment was not feasible and who failed a gemcitabine-based palliative frontline chemotherapy or adjuvant gemcitabine within 6 months were eligible for inclusion.

Additional eligibility criteria included the following: at least one measurable or evaluable lesion based on the response evaluation criteria in solid tumors (RECIST) 1.1, adequate bone marrow (absolute neutrophil counts [ANC] ≥ 1.5 × 10^9^/L, number of thrombocytes ≥ 100 × 10^9^/L), hepatic function (total bilirubin ≤ 1.5 × the upper limit of normal [ULN], or < 3.0 × ULN, in patients who underwent drainage procedure expecting normalization of the level, aspartate transaminase (AST) and/or alanine transaminase (ALT) ≤ 3 × ULN [in case of liver metastasis, 5 × ULN]), renal function (serum creatinine ≤ 1.5 mg/dL or creatinine clearance ≥ 50 mL/min), cardiac function (left ventricle ejection fraction ≥ 50% or age under 60 years old without symptoms), and patent biliary stent for at least 2 months. The following patients were excluded: patients who previously received irinotecan or oxaliplatin regimen, or any chemotherapy within 3 weeks prior to enrolment, had uncontrolled brain metastases, or other types of cancer except non-melanoma skin cancer, differentiated thyroid carcinoma, cervix carcinoma in situ, or peripheral neuropathy limiting life activities.

### Procedures

Attenuated FOLFIRINOX consisted of oxaliplatin 65 mg/m^2^, irinotecan 135 mg/m^2^, and leucovorin 400 mg/m^2^ injected intravenously on day 1 along with 5-FU 2000 mg/m^2^ continuous intravenous infusion over 46 h on days 1–2. Each cycle was planned to be repeated every 2 weeks. We could not obtain dose-finding phase I data for FOLFIRINOX. Accordingly, we planned the doses of all drugs in our attenuated FOLFIRINOX to be 75% of the dose in the Prodige 4/Accord 11 study [7] and removed the bolus 5-FU. The plan was for chemotherapy administration until observation of disease progression or unacceptable toxicity. This study design did not include any prophylactic granulocyte colony-stimulating factor or antibiotics.

Patient’s medical history, complete physical examination, neurologic examination, ECOG performance status, plain chest radiograph, complete blood counts, blood chemistry, and serum tumor markers were checked within 7 days prior to enrollment. Abdomen-pelvis computed tomography (CT) scan or magnetic resonance imaging (MRI), metastatic organ CT, or bone scintigraphy (in case of bone pain) was performed within 28 days prior to enrollment. Baseline assessment of patients’ status was repetitively performed within 3 days after every cycle’s commencement. Response evaluation with CT scan or MRI was performed every three cycles. Tumor response was assessed by investigators, based on RECIST v1.1. Toxicity profiles were determined using National Cancer Institute Common Terminology Criteria for Adverse Events, version 4.0.

### Statistical analysis

We used Simon’s 2-stage optimal design to determine the sample size. Assuming a PFS of 2.4 months (null hypothesis) with other 5-FU-based therapies and a target PFS of 4.3 months reflecting clinical activity of the FOLFIRINOX regimen, with an α error of 0.05, we calculated that a total of 45 patients would provide 80% power in detecting an effect on the primary outcome, assuming a 10% rate of dropouts or withdrawals. PFS and OS were computed using the Kaplan–Meier method with two-sided 95% confidence intervals (CIs). Censored subjects are indicated on the Kaplan–Meier curve as tick marks; these marks do not terminate the interval. Cox proportional hazards regression model was performed to identify prognostic factors to predict better PFS and OS. All tests were two-sided, and a *p* value < 0.05 was considered statistically significant. All analyses were performed using SPSS for Windows, version 19.0 (SPSS Inc, Chicago, IL, USA).

## Results

### Patients’ characteristics

Between October 2013 and February 2015, 41 patients participated and fulfilled the criteria for examining our hypothesis without dropout. Two of the 41 patients could not be evaluated; therefore, data on 39 patients were ultimately analyzed. Baseline demographic and clinical characteristics are shown in Table 1. The median age was 58 years (range 42–75 years). The numbers of men and women were 29 (74.4%) and 10 (25.6%), respectively. ECOG performance status was usually 0 or 1, except among two participants. A major protocol violation occurred involving two enrolled patients who each had an ECOG PS score of 2. The two patients completed scheduled chemotherapy without life-threatening complications and had evaluable outcomes; therefore, they were included in the final analysis. Seven patients (17.9%) had locally advanced unresectable disease, while other patients had metastatic disease. The most frequent metastatic site was the liver (43.6%), while 13 patients (33.3%) had multiple metastatic organ involvement. Twelve patients (30.8%) received 3rd-line palliative chemotherapy after being censored from the study. Median serum carbohydrate antigen 19-9 (CA19-9) and carcinoembryonic antigen (CEA) values were 864.0 IU/mL (2.0–400,000.0 IU/mL) and 10.3 U/mL (0.0–1386.0 U/mL) in 39 patients, respectively.

**Table 1:** Baseline demographic and clinical characteristics of 39 evaluated patients with gemcitabine-refractory advanced pancreatic cancer

Characteristic	No. of patients	%
Age		
≤ 60 years	25	64.1
> 60 years	14	35.9
Sex		
Male	29	74.4
Female	10	25.6
ECOG PS		
0	2	5.1
1	35	89.7
2	2	5.1
Extent of disease		
Locally advanced	7	17.9
Metastatic	32	82.1
Primary tumor location		
Head	18	46.1
Body	9	23.1
Tail	12	30.8
Metastatic site		
Liver	17	43.6
Lung	7	17.9
Distant lymph nodes	14	35.9
Peritoneum	7	17.9
Multiple organs	13	33.3
CA19-9 value (U/mL)		
> 10 times of UNL	21	53.8
≤ 10 times of UNL	18	46.2
Patients who received 3rd line chemotherapy	12	30.8

*ECOG PS* Eastern Cooperative Oncology Group Performance status, *CA19-9* carbohydrate antigen 19-9, *UNL* upper normal limit

### Treatment and outcomes

A total of 215 cycles were delivered to 39 patients with a median of 3 cycles/patient (range 1–17 cycles). The calculated median relative dose intensities (ranges) of 5-FU, oxaliplatin, and irinotecan were 76.4% (38%–100%), 79.5% (40.8%–100%), and 76.4% (40.6%–100%), respectively. The number and proportion of dose reductions and delays are summarized in Table 2, while the dose reduction protocol is presented in Table 3.

**Table 2:** Summary of dose reductions and delays

Characteristic	Number	%
Dose reductions and delays per patient		
Number of patients	39	
Dose delays	24	61.5
Dose reductions	11	28.2
Dose reductions and delays per cycle		
Number of cycles	215	
Dose delays	47	21.9
Dose reductions		
5-Fluorouracil	33	15.3
Oxaliplatin	39	18.1
Irinotecan	46	21.4

**Table 3:** Summary of events that resulted in dose reductions and delays

Toxicity	Grade	Oxaliplatin, 5-FU/Leucovorin	Irinotecan
Hemoglobin	Any grade	No reduction	
Neutropenia	3 (ANC 500–999/mm^3^)	No reduction (1 week delay)	
	4 (ANC ≤ 499/mm^3)^	25% reduction	25% reduction
Febrile neutropenia	3 (ANC < 1000/mm^3^ and body temperature ≥ 38.5 °C)	25% reduction	25% reduction
	4 (grade 3 febrile neutropenia and life threatening sepsis)	50% reduction	50% reduction
Thrombocytopenia	3 (25,000–50,000/mm^3^)	25% reduction	25% reduction
	4 (< 25,000/mm^3^)		25% reduction

*ANC* absolute neutrophil count

**Fig. 1:**
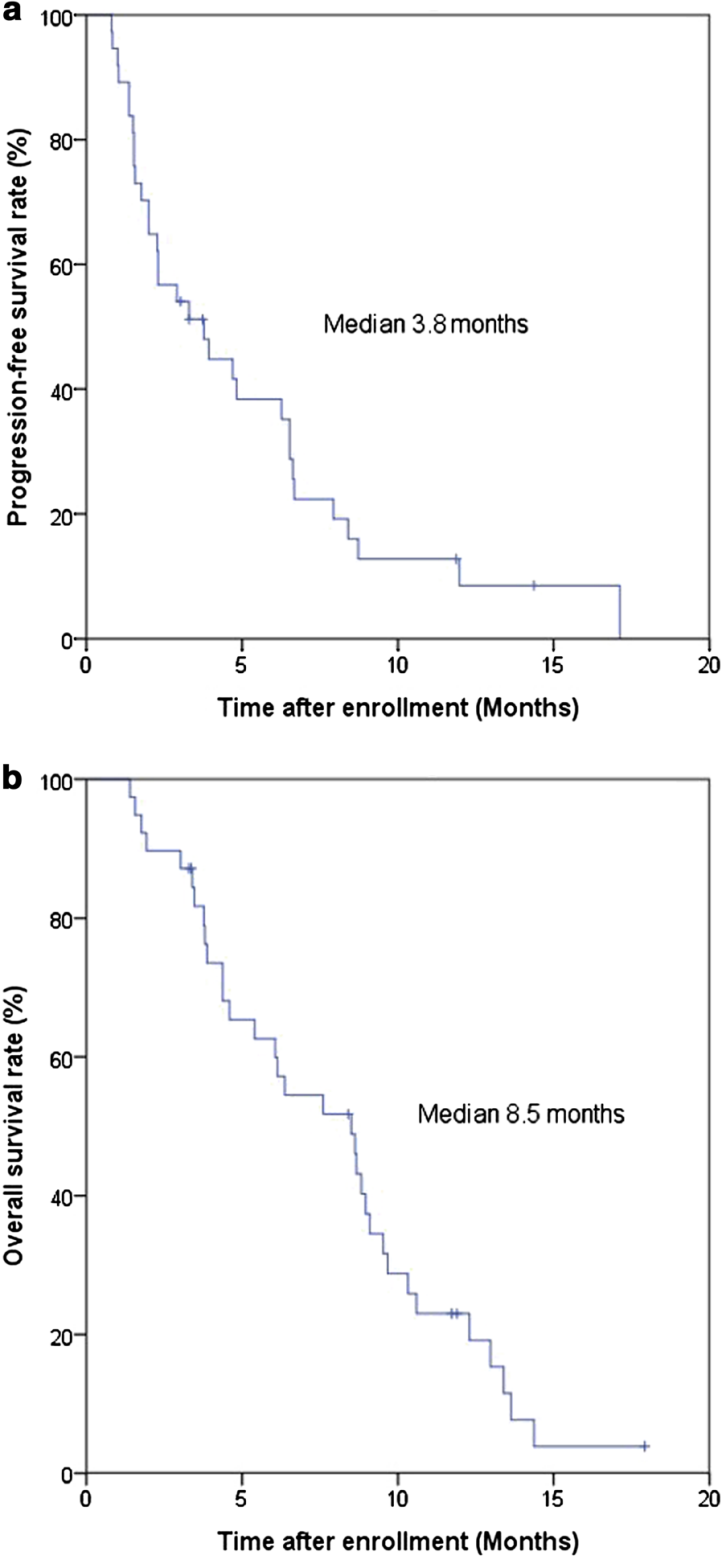
Kaplan–Meier curves of progression-free survival (PFS) and overall survival (OS) for 39 patients with pancreatic cancer. **a** The median PFS was 3.8 months (95% confidence interval [CI] 1.5–6.0 months). **b** The median OS was 8.5 months (95% CI 5.6–11.4 months)

Four patients achieved a partial response. Seventeen patients (43.6%) had stable disease, and 14 patients (35.9%) had progressive disease. The ORR was 10.3% (4 of 39 patients), and the DCR was 64.1% (25 of 39 patients). After a median follow-up period of 17.9 months, the median PFS was 3.8 months (95% CI 1.5–6.0 months) and the median OS was 8.5 months (95% CI 5.6–11.4 months). The 6-month and 1-year overall survival rates were 59.0% and 15.4%, respectively (Fig. 1). Multivariate analysis revealed no significant difference in PFS or OS according to age, sex, primary tumor location, liver metastasis, serum level of CA 19-9, presence of dose delays, or reductions of FOLFIRINOX (Table 4).

**Table 4:** Prognostic factor analysis of 39 patients treated with FOLFIRINOX as a second-line treatment

Variable	Progression-free survival			Overall survival		
	Median, months (95% CI)	Univariate (*p* value)	Multivariate hazard ratio (*p* value)	Median, months (95% CI)	Univariate (*p* value)	Multivariate hazard ratio (*p* value)
Age						
≤ 60 years	2.3 (0.89–3.71)	0.209	0.565 (0.193)	8.5 (3.39–13.61)	0.514	0.563 (0.209)
> 60 years	4.83 (2.15–7.51)			8.63 (5.58–11.69)		
Sex						
Male	3.3 (1.28–5.32)	0.827		8.5 (4.49–12.51)	0.854	
Female	3.73 (0–9.46)			8.83 (5.24–12.42)		
Extent of disease						
Locally advanced	1.77 (0–4.22)	0.465	1.318 (0.616)	9.67 (7.22–12.12)	0.14	0.379 (0.05)
Metastatic	4.7 (1.86–7.54)			6.37 (3.56–9.18)		
Primary tumor location						
Head	4.7 (0.51–8.89)	0.332	0.711 (0.399)	8.83 (5.55–12.11)	0.569	0.862 (0.719)
Body or tail	2.9 (1.44–4.36)			7.6 (1.74–13.46)		
Liver metastasis						
Yes	6.53 (2.04–11.02)	0.414	0.642 (0.29)	6.37 (2.94–9.8)	0.459	1.052 (0.897)
No	2.9 (0–6.19)			8.67 (8.18–9.16)		
CA19-9 value (U/mL)						
> 10 times of UNL	3.93 (1.52–6.35)	0.972		8.5 (5.51–11.49)	0.653	
≤ 10 times of UNL	3.77 (1.17–6.37)			8.83 (2.66–14.99)		

*CA19-9* carbohydrate antigen 19-9, *UNL* upper normal limit

### Toxicity data

The most common grade 3 or 4 toxicity was neutropenia (16/39, 41.0%), but no patient experienced febrile neutropenia. Nausea and anorexia (both 10.3%) were the second most common adverse events. Toxicity data are presented in Table 5. Septic shock with infection focus in the biliary tract occurred in a patient at 24 days after the 3rd cycle of FOLFIRINOX administration. The patient’s white blood cell count was 27,000/mm^3^ on admission. The patient died 11 days after presenting septic shock.

**Table 5:** Hematologic and nonhematologic adverse events which ever occurred in 39 patients treated with FOLFIRINOX as a second-line treatment

Adverse event	Any grade		Grade 3 or more	
	Number of patients	%	Number of patients	%
Hematologic				
Neutropenia	18	46.2	16	41.0
Thrombocytopenia	7	17.9	1	2.6
Anemia	23	59.0	3	7.7
Nonhematologic				
Nausea	23	59.0	4	10.3
Vomiting	3	7.7	1	2.6
Diarrhea	11	28.2	1	2.6
Fatigue	9	23.1	2	5.1
Anorexia	21	53.8	4	10.3
Constipation	6	15.4		
Mucositis	12	30.8	3	7.7
Neuropathy	5	12.8	1	2.6
Septic shock			1^a^	2.6
Pneumonia/pleural effusion			2	5.1
Abdominal distension			1	2.6
Alkaline phosphatase increased			1	2.6
Hypokalemia			1	2.6
Glossopharyngeal neuralgia			1	2.6

^a^Grade 5 (death)

## Discussion

The present study prospectively investigated the safety and efficacy of an attenuated dose of FOLFIRINOX as a second-line therapy for advanced pancreatic cancer and showed encouraging length of progression-free survival and overall survival, but was accompanied by significant toxicity.

Although several analyses on the efficacy and safety of modified doses of FOLFIRINOX have been performed, conclusive evidence is still lacking. Ghorani et al. [16] retrospectively analyzed data from their single-center experience in the United Kingdom with modified FOLFIRINOX for chemotherapy-naïve advanced pancreatic cancer. The treatment regimen consisted of oxaliplatin 85 mg/m^2^, irinotecan 130–135 mg/m^2^, folinic acid 400 mg/m^2^, and 5-FU infusion 2400 mg/m^2^ over 46 h, administered every 14 days. Their study included 15 patients with stage IV disease and indicated 47% ORR, with median PFS and OS of 7.2 and 9.3 months, respectively. The authors reported grade 3 or 4 neutropenia in 5.6% of their patients and non-hematologic adverse events (grade 3 or 4 vomiting: 27.8%). Blazer et al. [17] and Lakatos et al. [18] tested modified FOLFIRINOX in patients with locally advanced disease. Dose intensities varied between the research groups, but these studies reported a relatively low rate of adverse events along with meaningful disease control.

Umemura et al. [19] used a modified dose of FOLFIRINOX to treat a small group of patients with unresectable locally advanced or metastatic pancreatic cancer who failed on gemcitabine- and S-1-based therapies. The authors designed the modified regimen with oxaliplatin 75 mg/m^2^, irinotecan 150 mg/m^2^, folinic acid 320 mg/m^2^, 5-FU bolus 320 mg/m^2,^ and 5-FU infusion 1920 mg/m^2^ administered every 21 days. After a total of 114 cycles were delivered in 13 patients, the ORR obtained was 30.8% with no CR, and median PFS and OS were 137 and 176 days, respectively. Five patients (38.5%) experienced grade 3 or 4 neutropenia, but febrile neutropenia did not occur. The other common grade 3 or 4 adverse events were anorexia, vomiting, and diarrhea (3, 1, and 1 patients, respectively). The incidence of grade 3–4 diarrhea in clinical trials in the Republic of Korea has been low compared with that reported in Western trials. Similar incidence is reported in Japanese patients. Ethnic differences in the activity of enzymes that metabolize irinotecan appear to be the cause. In a phase II trial of modified FOLFIRI.3 compared with modified FOLFOX as a second-line treatment for advanced pancreatic cancer conducted by Yoo et al. [11] in Korea, irinotecan dose was 140 mg/m^2^ every 2 weeks, and grade 3–4 diarrhea was observed in 7% of 31 patients. Kobayashi et al. [20] reported no occurrence of grade 3–4 diarrhea in 18 Japanese patients who were enrolled in a prospective phase I/II study of FOLFIRINOX as a second-line treatment for metastatic pancreatic cancer. The phase I trial was a dose-finding study for irinotecan in combination with fixed-dose 5-FU, leucovorin and oxaliplatin. As a result, an irinotecan dose of 100 mg/m^2^ was recommended. This finding is consistent with the frequent dose reduction of irinotecan in this study. The results of those two trials are very similar to those of the current study.

In a small retrospective analysis of salvage treatment with FOLFIRINOX, Lee et al. [13] reported a DCR of 55.6%, with median PFS and OS of 2.8 and 8.4 months, respectively. These findings are similar to the outcomes obtained with our attenuated dose of FOLFIRINOX, although the FOLFIRINOX regimen reported by Lee et al. consisted of the same dose as that of the original PRODIGE/ACCORD 11 trial. Clinical outcomes of several studies with modified FOLFIRINOX as a frontline treatment appeared not to be inferior to those of the original dose [21, 22]. In the CONKO-003 trial, which confirmed the survival benefit of salvage chemotherapy described above, the median OS was 5.9 and 3.3 months, and time to progression was 2.9 and 2.0 months (OFF versus FF, respectively). The efficacy of OFF regimen was not significantly different from that of our attenuated FOLFIRINOX in terms of PFS but tends to be inferior in terms of OS. In this previous study, 25% of the subjects received third-line therapy, similarly to our series of patients. The results of a few single-arm studies with irinotecan, fluorouracil, and leucovorin (FOLFIRI) as a second-line chemotherapy for pancreatic cancer have also been published. Zaniboni et al. [10] reported that 36% of patients showed DCR, with median PFS and OS of 3.2 and 5 months, respectively, using a FOLFIRI regimen in the GISCAD multicenter phase II study. Yoo et al. [12] stated that DCR was achieved in 23% of patients, with a median OS of 3.9 months in response to their modified FOLFIRI.3 chemotherapy in a randomized phase II study. These outcomes suggest the need for a triple combination of drugs for gemcitabine-refractory pancreatic cancer, despite concerns regarding their higher toxicity.

Attenuated FOLFIRINOX therapy in our study achieved a meaningful OS benefit, although the ORR fell short of our expectations. However, this dose-modified FOLFIRINOX regimen did not reduce toxicity either. Forty-one percent prevalence of grade 3 toxicities or neutropenia and an event of treatment-related mortality still leave concerns regarding the safety of this regimen. The small size of the study population for subgroup analysis, the absence of biomarker evaluation, and the lack of an assessment of the quality of life are also limitations of the present study. There is increasing interest in the feasibility and safety of FOLFIRINOX as a second-line treatment. Before initiation of our study, gemcitabine-based front-line therapy was doubted for its survival benefit because only modest effects were observed when gemcitabine in combination with erlotinib was compared with gemcitabine alone.

Recently, a new regimen of albumin-bound paclitaxel (nab-paclitaxel) plus gemcitabine was shown to be superior to gemcitabine alone for OS as a front-line treatment in a randomized phase III study; thus, nab-paclitaxel plus gemcitabine is becoming one of the most widely used options as a first-line treatment [23–26]. Accordingly, the importance of salvage chemotherapy, which involves neither gemcitabine nor taxane, is increasing.

The higher DCR and longer OS observed in the current study justify the triple combination of FOLFIRINOX over doublet regimens such as FOLFIRI or FOLFOX, even in heavily pretreated pancreatic cancer patients. To date, only a handful of regimens apart from FOLFIRINOX could obtain a median OS beyond 8 months from initiation of second-line therapy for advanced pancreatic cancer [26]. Based on these findings, a phase III trial to confirm the survival benefits of modified FOLFIRINOX as a second-line treatment for advanced pancreatic cancer is being planned.

## Conclusions

FOLFIRINOX is not only a front-line treatment of choice for advanced pancreatic cancer but can also be a promising option as second-line therapy for gemcitabine-refractory pancreatic cancer. An attenuated dose of FOLFIRINOX does not diminish its efficacy; however, careful selection of patients is required to avoid its high toxicity.
